# A Silent Infection Pandemic of COVID-19: Epidemiological Investigation and Hypothetical Models

**DOI:** 10.1155/2020/5120253

**Published:** 2020-07-05

**Authors:** Jianping Geng, Jun Yu, Tao Lu, Yinhe Wang, Yang Cao

**Affiliations:** ^1^Xi'an International Medical Center Hospital, Xi'an 710100, Shannxi, China; ^2^Nanjing Drum Tower Hospital Affiliated to Nanjing University School of Medicine, Nanjing 210008, China; ^3^Clinical Epidemiology and Biostatistics, School of Medical Sciences, Örebro University, Örebro 70182, Sweden

## Abstract

To explore the epidemic mode of COVID-19, we made an epidemiological investigation, set up hypothetical models, and compared them with hepatitis A virus (HAV) age-specific epidemic characteristic. In the epidemiological investigation, we reported the first familial COVID-19 silent infection in the world. A 19-year-old healthy female COVID-19 virus carrier without any symptoms caused two mild and one severe pneumonia. In hypothetical models, the silent infection rate ranges from 60% to 80% based on 3 sources: China mainland, evacuation of 4 nationals, and the ship “Diamond Princess,” respectively. In comparison with HAV, COVID-19 shows the same infection mode in children (aged 0–9 years), but significant difference in young adults (aged 10–44 years) and the elderly (aged 45 years or older). Therefore, we prejudged that COVID-19 is a silent infection pandemic mainly in young adults but threatens the elderly.

## 1. Introduction

The coronavirus disease 2019 (COVID-19), caused by the severe acute respiratory syndrome coronavirus 2 (SARS-CoV-2), has resulted in a pandemic and poses great public health threat all over the world [1]. Until June 19, 2020, the number of confirmed COVID-19 cases has been over 8,578,000, with 456,000 deaths. The most severe situation happened in China, Italy, USA, Spain, Germany, and Korea. The United States has the largest number of COVID-19 cases, and the overall case-fatality rate (5.3%) is substantially higher than that in Italy, which causes widespread concern [2]. By conducting an epidemiological investigation, exploring the hypothetical models, and comparing the age-specific epidemic characteristics of COVID-19 with those of hepatitis A virus (HAV), we explored the mode of COVID-19 transmission and spread.

## 2. Epidemiological Investigation: An Asymptomatic COVID-19 Carrier Caused a Family Cluster with One Severe and Two Mild Pneumonia

The index is a 19-year-old girl who studied in a university in Wuhan and returned to her hometown Anyang, in Henan Province, on January 10, 2020. She had no fever, sore throat, or any respiratory symptoms for 60 days till March 9, the end of our follow-up. However, her three family members closely living with her were infected by COVID-19. On January 26, 2020, she was isolated without any symptoms and was negative for both chest X-ray examination and COVID-19 nucleic acid test (NAT) (Sansure Biotech, Changsha, China) using throat and nose swab samples. The girl had no diseases and drug history recorded recently. The Anyang Municipal Center for Disease Control and Prevention (CDC) tested the girl again on January 28, 2020, and both throat and nose swab samples were positive for COVID-19 NAT. It had been 19 days since the girl came back home, which exceeded China CDC's current maximum incubation period of 14 days [3]. On February 1, 5, and 8, the girl was tested repeatedly, and all the results were negative.

Patient 1, female, aged 47, the girl's young aunt, went to the clinic for treatment due to fever and sore throat on January 14, 2020. The symptoms were mitigated after taking medication. However, the symptoms appeared again and worsened on January 24. The woman was isolated for treatment and diagnosed as COVID-19 infection on January 26. And the throat swab and sputum samples collected from the woman were positive for COVID-19 NAT on the same day.

Patient 2, male, aged 45, the girl's father, had a fever and respiratory symptoms on January 23 and was isolated for treatment in the Anyang People's Hospital on January 26. He was diagnosed as a suspected case of pneumonia with COVID-19 infection by an expert group consultation. The Anyang Municipal CDC tested the throat swab and sputum samples collected from the patient on January 26, and both the results were positive for COVID-19 NAT.

Patient 3, female, aged 48, the girl's old aunt, had a fever and respiratory symptoms occurred on January 25. After isolation for treatment and consultation by an expert group, the woman was diagnosed as a suspected case of pneumonia with COVID-19 infection. Her throat swab and sputum samples were positive for COVID-19 NAT on January 26.

**Figure 1:**
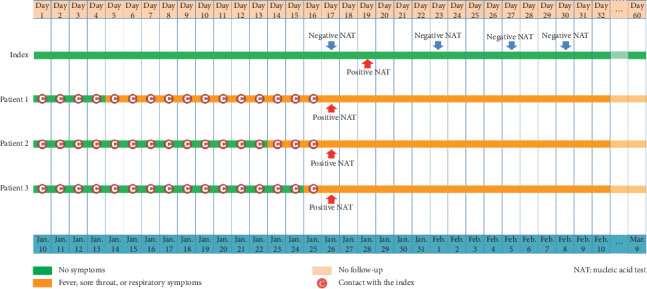
Timeline of exposure to the asymptomatic index with COVID-19 infection in Anyang, Henan, China.

The three patients had no travel and living history in Wuhan and only had contact with the index. Other potential infection sources were excluded by the Anyang Municipal CDC's track history investigation. The index and three patients with confirmed COVID-19 infection were admitted to the Anyang No. 5 People's Hospital for clinical monitoring and isolation. Patient 1 showed severe pneumonia, but patients 2 and 3 showed mild clinical manifestation. The timeline is shown in Figure 1.

## 3. Epidemiological Theoretical Models

### 3.1. Model 1

According to the data released by China's CDC on February 10, 2020, there were 7,333 severe cases and 37,626 confirmed cases [4]. In our silent infection hypothetical model, the proportion of apparently confirmed infection in the total number of infection was equal to the proportion of severe cases in the confirmed cases. The hypothetical formula is as follows:

(1) $$\begin{array}{c} \frac{\text{confirmed}}{\text{total infection}}=\frac{\text{severe}}{\text{confirmed}}. \end{array}$$

That is,

(2) $$\begin{array}{c} \frac{\text{total}-\text{silent}}{\text{total infection}}=\frac{7333}{37626}. \end{array}$$

Therefore,

(3) $$\begin{array}{c} \frac{\text{silent infection}}{\text{total infection}}=80.5\text{\%}. \end{array}$$

According to our mathematical models of epidemic data in the early of February 2020 in Mainland China, silent infection is estimated to account for as many as 80.5% of the total COVID-19 infections.

Our hypothetical model suggests that severe cases may only account for about 4% of the total number of infections if COVID-19 could be effectively prevented and controlled in the early stage, which is bearable when the hospital resources are sufficient. However, if the early prevention and control measures of the epidemic failed, it might cause cluster transmission within families and serious delay of mild illness, and the number and proportion of severe cases would increase exponentially, which might soon exceed the hospital's saturated carrying capacity. As what happened in March 2020, the epidemic situation in Italy and Spain exceeded the hospital's saturation capacity and required external help.

### 3.2. Model 2

According to the information obtained from the ship Diamond Princess on February 18 in Yokohama, Japan, there were 88 newly confirmed cases of infection, 65 of which were asymptomatic. Based on a rough estimation, asymptomatic infection accounted for 73.9% of the total number of confirmed infections. This proportion happened after 2 to 3 weeks since the ship was isolated and is basically consistent with our estimation in model 1.

### 3.3. Model 3

From 29 to 31 January, Japan, South Korea, Singapore, and Germany withdrew their overseas citizens from Wuhan, separately. After the evacuations, these overseas citizens were all received rigorous physical examination. Five of 368 withdrawn Koreans were confirmed as COVID-19 infected. The infection rate was 1.37%. Two of 124 withdrawn Germans were confirmed infectees. The infection rate was 1.61%. One of 92 withdrawn Singaporeans was an infectee, and the infection rate was 1.08%. Eight of 565 withdrawn Japanese were infectees with an infection rate of 1.42%. The infection rates of evacuations in these four countries ranged from 1.08% to 1.61%. The overall infection rate of the evacuated 1,149 citizens of the four countries is 1.39%.

During the Chinese Spring Festival, 9 million people lived in Wuhan. Based on the above-estimated infection rate (1.39%), about 125,100 people might have been infected by COVID-19. According to this calculation, the proportion of asymptomatic infections in the total infections was 69.9% with a range from 61.3% to 74.1%.

Based on the above three epidemiological theoretical models, the silent infection rate of COVID-19 infection may range from 60% to 80%, which suggests that the COVID-19 is mainly silent infection pandemic.

## 4. Comparison of Age Distribution between COVID-19 and Hepatitis A Virus (HAV)

HAV also has a very high silent infection rate. In the era without vaccines, almost every person would get hepatitis A (HA). It was very dangerous if only a few persons in the population were infected with HAV. HA would become an epidemic after most of the population was infected with HAV. The typical incident was the HA pandemic in Shanghai in 1988 [5].

COVID-19 is also in the same situation. If there is no immune barrier for the population, whether it is natural immunization or vaccination, a pandemic is inevitable. After at least half of the urban population was infected, the pandemic would naturally stop if there was no isolation, disinfection, and/or other interventions.

The infection rate consists of both apparent and silent infections. The HAV pandemic in Shanghai in 1988 represented a normal distribution of age with a peak among people 20 or 30 years old. COVID-19 also represented similar distribution, although there were no confirmed epidemiological data on antibody distributions available currently.

According to the current epidemic data of COVID-19 [6–9], children between 0 and 9 years old accounted for only 1% of the total infected population with a mortality rate of 0. Although the children were infected with the HAV in the HA pandemic in Shanghai, most of them were silent infections and no children died [5]. In this situation, it is actually equivalent to immune protection among the children infected with the virus.

**Figure 2:**
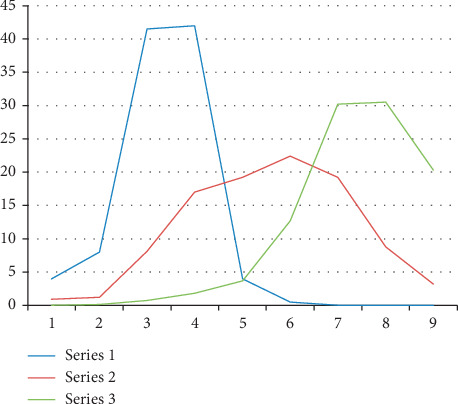
The comparison between HAV and COVID-19. The age-specific distribution of HAV morbidity rate in 1988 in Shanghai (series 1), COVID-19 morbidity rate (series 2), and COVID-19 death rate (series 3) in China.

According to the epidemic data of COVID-19 for age distribution released by China CDC on February 24, 2020 (Figure 2), the average age of patients was 51 years, and 77.8% of patients were aged 30–69 years. The age distribution of COVID-19 infectees was nonnormal with a median of 51 years. Compared with the normal distribution of infection rates with a mean of 30 years, the average age of infectees was actually older. Among patients infected with HAV in Shanghai, patients aged 20–39 years accounted for 83.46% of the total cases. Patients over 50 years accounted for less than 1% [10].

Young adults infected by HAV had the highest mortality, due to the acute hepatitis characterized with a cytokine storm. Not only did the elderly account for a small proportion, the mortality rate was also very low, which was approximated to 0. However, the elderly patients with COVID-19 had the highest mortality. With age increasing, the mortality rate rises almost linearly. About 92% of the deaths were over 50 years old. The comparison between HAV and COVID-19 is shown in Figure 2.

To summarize, the COVID-19 and HA are both silent infection pandemics. Although there were similar proportions of incidence and death in children due to the protection effects of silent infection, there was an obvious difference in middle-aged people between COVID-19 and HA. COVID-19 was more prevalent among young people, but mostly affected the middle-aged and the old people.

## 5. Discussion

In Segen's Medical Dictionary, silent infection is defined as an infection lacking significant clinical signs of disease. Silent infections may be recognized only in retrospect, e.g., by a 4-fold increase in antibody titres to a particular pathogen, especially viruses. McGraw-Hill Concise Dictionary gives a similar definition. In our article, silent infection includes both confirmed asymptomatic patients and not confirmed with viral shedding.

Our epidemiological investigation shows that COVID-19 silent infection transmission may result in not only mild clinical illness but also severe clinical illness. It implies that COVID-19 infection power is quite strong. There is also a Germany report about asymptomatic carriers, but the final result is unspecific symptom patient [11, 12]. Our epidemiological investigation lasted for 2 months and the index showed no symptom. The silent infection transmission spreads widely and rapidly. It quickly occupied Wuhan and then occupied other 13 cities in Hubei province, in China. Now, it has occupied almost all the countries in the world.

The proportion and age-specific distribution of silent infection are very important epidemic characteristics of COVID-19. Our hypothetical models and comparison with HAV indicate that silent infection plays major infection of COVID-19 and focuses on young adults. Meanwhile, the data before January 23, 2020, in Wuhan, China, were also analyzed by a networked dynamic metapopulation model and Bayesian inference [13, 14]. In the analysis, undocumented cases accounted for 86% of the total infection cases. The result is consistent with those based on our hypothetical model.

The effective prevention and control for apparent infection is early detection and early isolation. In contrast, the prevention and control for silent infection is overall isolation and overall disinfection. These prevention and control measures are significantly different. The prevention and control measures of COVID-19 belong to the later. Overall isolation and overall disinfection should be implemented as soon as possible. These measures are the key for the prevention and control of COVID-19.

The successful prevention and control of COVID-19 in other provinces (except Wuhan and Hubei provinces) in China also confirmed that these “hardcore” measures are indeed very effective. Most of the provinces in China controlled the COVID-19 epidemic within 2–4 weeks. However, the similar measures of overall isolation in Wuhan and Hubei province continued 2-3 months until the epidemic exponential growth curve passed the turning point. The prevention and control measures in Korea followed overall disinfection but not strictly overall isolation; therefore, it presented a persistent low-level epidemic. However, Italy, Spain, and New York had no isolation and no disinfection at the early stage; therefore, severe outbreak presented in these countries.

After total isolation, the apparent infectors would disappear. However, the asymptomatic patients still exist. For example, Wuhan tested all citizens after isolation from May 16 to 30, which found about 300 asymptomatic patients in 10 million residents. If the prevention was relaxed, the epidemic would rebound in the city. However, if all residents still wear masks always and wash hands often, and the monitoring work is still running well, there will only be sporadic outbreak, but no epidemic. For sporadic outbreak, we may only take simple measures such as local isolation.

## Data Availability

The data used to support the findings of this study are included within the article.
